# Simian virus 40 vectors for pulmonary gene therapy

**DOI:** 10.1186/1465-9921-8-74

**Published:** 2007-10-29

**Authors:** Luminita Eid, Zohar Bromberg, Mahmoud Abd EL-Latif, Evelyn Zeira, Ariella Oppenheim, Yoram G Weiss

**Affiliations:** 1 Department of Anesthesiology and Critical Care Medicine, Hadassah – Hebrew University Medical Center, Jerusalem, 91120, Israel; 2 The Goldyne Savad Gene Therapy Institute, Jerusalem, 91120, Israel; 3 Department of Hematology, Hadassah – Hebrew University Medical Center, 91120, Jerusalem, Israel; 4 Department of Anesthesiology and Critical Care, University of Pennsylvania School of Medicine, Philadelphia, PA 19104-4283, USA

## Abstract

**Background:**

Sepsis remains the leading cause of death in critically ill patients. One of the primary organs affected by sepsis is the lung, presenting as the Acute Respiratory Distress Syndrome (ARDS). Organ damage in sepsis involves an alteration in gene expression, making gene transfer a potential therapeutic modality. This work examines the feasibility of applying simian virus 40 (SV40) vectors for pulmonary gene therapy.

**Methods:**

Sepsis-induced ARDS was established by cecal ligation double puncture (2CLP). SV40 vectors carrying the luciferase reporter gene (SV/*luc) *were administered intratracheally immediately after sepsis induction. Sham operated (SO) as well as 2CLP rats given intratracheal PBS or adenovirus expressing luciferase served as controls. Luc transduction was evaluated by *in vivo *light detection, immunoassay and luciferase mRNA detection by RT-PCR in tissue harvested from septic rats. Vector abundance and distribution into alveolar cells was evaluated using immunostaining for the SV40 VP1 capsid protein as well as by double staining for VP1 and for the surfactant protein C (proSP-C). Immunostaining for T-lymphocytes was used to evaluate the cellular immune response induced by the vector.

**Results:**

Luc expression measured by *in vivo *light detection correlated with immunoassay from lung tissue harvested from the same rats. Moreover, our results showed vector presence in type II alveolar cells. The vector did not induce significant cellular immune response.

**Conclusion:**

In the present study we have demonstrated efficient uptake and expression of an SV40 vector in the lungs of animals with sepsis-induced ARDS. These vectors appear to be capable of *in vivo *transduction of alveolar type II cells and may thus become a future therapeutic tool.

## Background

Sepsis is the leading cause of death in critically ill patients [1]. Despite advances in treating the sepsis syndrome, the incidence and mortality of sepsis remains high (35–45%) [2]. Lung is the organ most often involved, with lung injury taking the form of Acute Respiratory Distress Syndrome (ARDS) [3-5], carrying 40% mortality [6]. The pathological hallmark of ARDS is airspace flooding with proteinaceous fluid, basement membrane disruption, hyaline membrane deposition, surfactant depletion, interstitial swelling, and interstitial neutrophilic infiltration [7]. Histological sections of the lungs from patients dying of ARDS and from animal models of the disease reveal: interstitial edema, followed by extensive necrosis of alveolar epithelial cells [8].

Alveolar epithelium in the adult lung consists of two cell types: Type I, differentiated cells that facilitate gas exchange and Type II, metabolically active cells involved in surfactant secretion and epithelial repair, serving as progenitors for injured type I cells [9]. Injury to type II cells impairs gas exchange by reducing the surfactant supply and limits type I cells regeneration, their preservation being essential for recovery from ARDS [10].

Nowadays therapy for ARDS remains mainly supportive, designed to prevent secondary injury [11]. However, recent studies demonstrated that organ damage in sepsis involves an alteration in gene expression. Decreased transcription of surfactant proteins [12] as well as profound pulmonary epithelial dysregulation together with altered levels of Hsp70 were found in animal models of ARDS [13]. We also showed in our previous studies that severe sepsis induced by cecal ligation and puncture (2CLP) precipitates ARDS [13,14]. Furthermore, we demonstrated that enhanced Hsp70 expression in pneumocytes using an adenoviral vector (AdHSP) not only decreased histological abnormalities in the lung but also improved short-term outcome [15]. Specifically, we showed that AdHSP limited sepsis-induced acute inflammation by suppressing NF-κB activation [50].

One approach to correct deficient protein expression is to use viral mediated gene transfer [16]. An optimal vector for gene therapy should be safe, efficient, nonimmunogenic, and available in high titers [17]. There is currently no single vector with all these advantages. SV40 based vectors are efficient gene delivery vehicles for a wide spectrum of *ex vivo *and *in vivo *targets, including hematopoetic, liver and kidney cells [18,19]. The vector was shown to be potentially effective in a number of clinical models, including Crigler Najjar syndrome [20], HIV, [21] cancer immunotherapy, [22-25], liver cirrhosis [26].

SV40 evades host immune response most likely by caveolar endocytosis, followed by vesicular transport to the endoplasmic reticulum [27], thus avoiding the more common endosomal-lysosomal pathway used by most viruses [28]. SV40 vectors were found to be nonimmunogenic, allowing repeated administrations and long survival of transduced cells [29,30]. Replication incompetent SV40 vectors in which the viral T-antigen is replaced by the gene of interest are produced *in vivo *in cells that supply T-antigen in trans-, such as COS or COT cells [31], and high vector titers may be readily prepared [32]. Cloning capacity of these vectors is limited; nevertheless, many potentially therapeutic genes may be accommodated, as shown by the wide spectrum of applications already investigated.

The use of SV40 vectors for gene transfer to the lung has not been explored. In this work we found that SV40 vectors may be used for lung cell transduction. After *in vivo *vector delivery, we established and measured expression of the reporter gene in rats with sepsis induced ARDS. Our results lead the way for studies regarding gene delivery to the lung, in order to modulate pulmonary disease processes.

## Methods

### Vector Construction

SV/*luc *is a T-antigen replacement vector carrying the firefly luciferase (*luc) *reporter gene [33]. Preparation of the SV/*luc *vector was performed as previously described [34]. Recombinant E1, E3-deleted adenoviral vectors (Ad/*luc) *were propagated in HEK293T cells, by commonly used methods[35].

### Induction of Sepsis/ARDS and Vector Administration to Rats

Animal procedures were approved by the Institutional Animal Care Ethical Committee. Under Ketamine/Xylasine/Isoflurane anesthesia, severe sepsis was induced in Sprague-Dawley rats using cecal ligation double puncture (2CLP) [14]. 1.25 × 10^8 ^IU/ml (infectious units) of SV/*luc *in 300 μl PBS were administered via a tracheal catheter to 2CLP and sham operated (SO) rats immediately after the procedure and to unoperated (UO) rats. In the positive control group, 10^9 ^IU/Ad/*luc *in 300 μl PBS were administered in the same way to 2CLP animals [15]. Negative control animals received PBS only.

### *In vivo *Light Detection

The reporter gene used encodes the luciferase protein, an enzyme that converts luciferin in presence of oxygen and ATP to a bioluminescent substance. *In vivo *light detection was performed using the Roper Chemiluminescence Imaging System, (CCCD-cooled coupled charged camera) model LN/CCD-1300EB equipped with ST-133 controller and a 50 mm Nikon lens (Roper Scientific, Princeton Instrument, Trenton, NJ). The system enables detection of an internal light signal emerging from mammalian tissue. The measurement is the sum of the integrated light signal subtracted the background light emission of an area of equal size [36]. A pseudo color image represents light intensity spectrum (from blue – least intense, to red – most intense).

Forty eight hours after vector administration, rats were reanesthetized. 125 mg/kg Beetle Luciferin (Promega, Madison, WI) was administered intraperitoneally, and luciferase activity was measured by photographing the animals first in light (to obtain the animal's image) and then in the dark (to measure light emission). A composite photograph was obtained by superimposing the two images. The average amount of fluorescence measured in light units per area, as detected by the CCCD camera represents signal intensity.

### Tissue Preparation

After the light emission measurement, at 48 hrs, the animals were sacrificed and internal organs harvested. One lung was removed, preserved in formalin, embedded in paraffin, cut at 5 μm thickness and stained with hematoxylin and eosin. H&E sections were evaluated for lung injury, degree of injury and its distribution within the lungs [14]. Part of the spleen, liver, heart and one kidney were also harvested and prepared in the same way. The remaining part of the spleen, heart, liver, the second kidney and lung were frozen in fluid nitrogen and preserved at -80°C for RNA.

### Immunohistochemical Detection

Immunostaining was performed using the procedure described by Lavon et al [37]. For luciferase we used a primary rabbit anti-mouse polyclonal antibody (Cortex, San Leandro, CA) at 1:50 dilution, followed by a secondary goat anti-rabbit IgG antibody (biotin conjugated, Zymed, San Francisco, CA). VP1 capsid protein detection was performed using a rabbit anti-SV40 polyclonal primary antiserum [38] followed by FITC-conjugated goat anti-rabbit IgG secondary antiserum (Zymed). DAPI (blue) was used as nuclear counterstain. Double immunostaining for surfactant protein C (ProSP-C) and VP1 was performed in order to detect internalization of VP1 viral capsid protein into the alveolar cells. Serial lung sections were immunostained for VP1 using the same rabbit polyclonal antibody as above followed by goat anti-rabbit Cyte 5, and for ProSP-C using rabbit anti-ProSP-C polyclonal primary antiserum (Alomone Inc, Israel) followed by FITC-conjugated goat anti-mouse IgG. Immunohistochemical detection of Lymphocyte (CD3+ T cells) infiltration was performed using the immunoperoxidase avidin biotin methodology. A primary CD3+ T cell antibody (monoclonal mouse anti-rabbit, affinity purified, Biosource, Carlsbad, California) at 1:100 dilution was used, followed by a secondary goat anti-rabbit IgG antibody (biotin conjugated, Zymed).

### Reverse Transcription – RT-PCR

Total RNAs from the frozen tissues were extracted using Trireagent (Sigma, Saint Louis, MO) according to the manufacture's instructions. A quantity of 2.5 μg of extracted RNA (for each sample) was subjected to reverse transcription using 400 u/μl RNAse, 0.5 μg/μl oligo dT, 200 u/μl M-MLV RT and 2.5 mM dNTPs. 2 μl of the reacting cDNA products were used as template for PCR. PCR was performed using PCR primers specific for Luciferase: 5'-TGGTCTGCCTAAAGGTGTCG-3' (forward) and 5'-ATGTAGTCTCAGTGAGCCC-3' (reverse), and carried out for 35 cycles. pGL3 Luciferase reporter vector (Promega) served as a positive control. GAPDH was used as a house keeping control gene, using PCR primers specific for GAPDH: 5'-ACCACAGTCCATGCCATCAC-3' and 5'-TCCACCACCCTGTTGCTGTA-3'.

## Results and Discussion

### Sepsis induces ARDS

In the present experiment, ARDS was induced secondary to intra-abdominal sepsis (2CLP). Starting 12 hours after 2CLP, and more prominently at 24 and 48 hours, 2CLP rats displayed the typical signs of sepsis: intense pallor/cyanosis of the mucous membranes, dirty fur with erected hairs, distended abdomen, diarrhea, tachypnea (40–60 breath per minute). Animal behavior was grossly abnormal: periods of agitation followed by periods of sleepiness along with limited movements and inability to feed.

**Figure 1:**
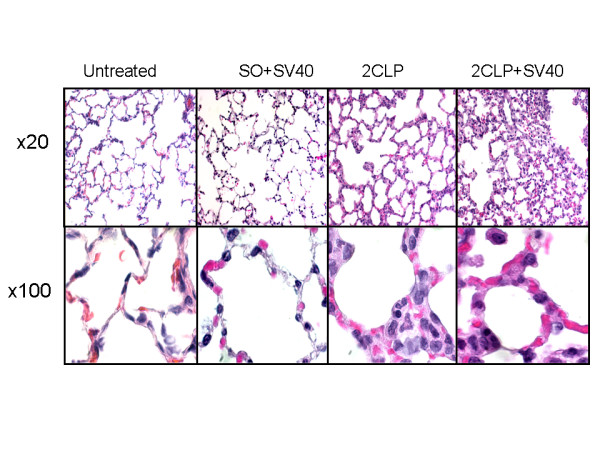
**Lung pathology**. H&E stained lung tissue, shown at ×20 and ×100 magnifications. Untreated control rats show normal lung histology; SO rats given intratracheal SV/*luc *also show normal histological appearance; 2CLP rats given intratracheal PBS or SV/*luc *show distinct ARDS pathology: alveoli filled with proteinaceous fluid, septal thickening, interstitial lymphocytic and neutrophilic infiltration.

Histological examination of the lung sections from 2CLP animals showed changes consistent with ARDS. Macroscopically: lungs were less aerated, covered with white fibrin patches and pleural fluid was found in different quantities. H&E stained sections depicted: alveoli filled with proteinaceous fluid, septal thickening, and interstitial neutrophilic infiltration (Figure 1). Mortality was present only in the septic (2CLP) animals. The 48 hrs mortality rate following 2CLP was similar to previously published numbers in sepsis induced ARDS [13-15].

### Use of SV40 vectors in ARDS

SV/*luc *or Ad/*luc *vectors were directly administered into the trachea of 2CLP and SO rats, immediately following the procedure. Using this route we achieved maximal vector concentration and distribution to the lung, limiting the systemic spread. Histopathologically there was no difference between the lungs of the septic rats given PBS or SV/*luc *(Figure 1). Mortality rate was similar in 2CLP animals given either PBS or SV/*luc*, suggesting that the vector itself did not add to the sepsis/ARDS induced mortality.

### Sepsis-induced ARDS increases Luciferase expression

At 24 and 48 hours, all the animals were reanesthetized and photographed with the CCCD camera to detect light emission. At 48 hours all animals were sacrificed and the lungs were harvested.

**Figure 2:**
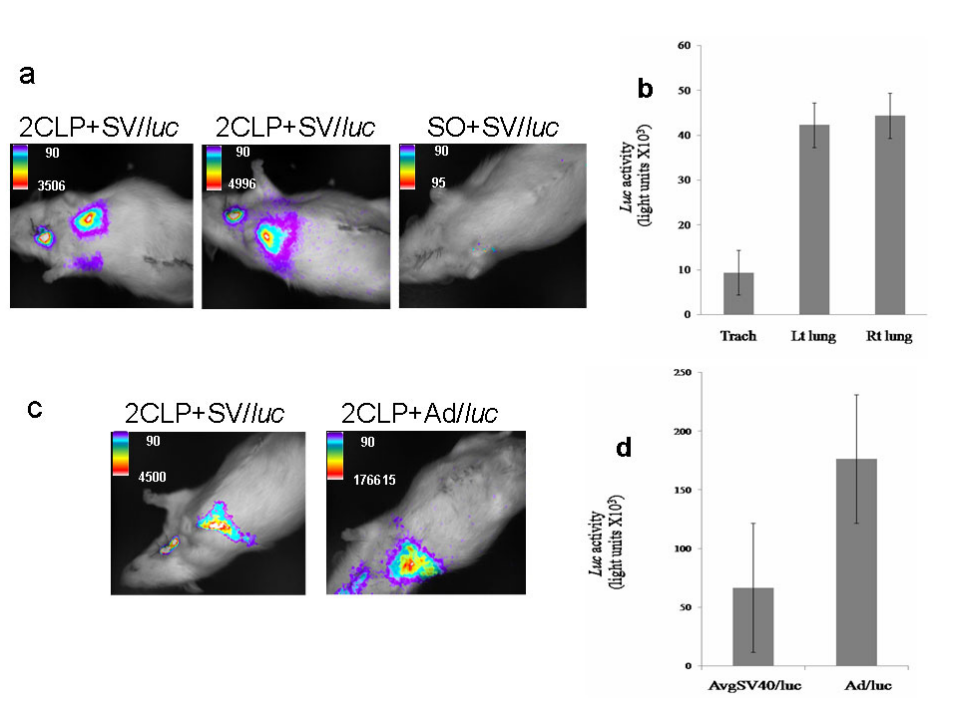
***In vivo *detection of luminescence**. Detection was performed with a CCCD camera (exposure time 2 min) 48 hours following intratracheal administration of 300 μl of 1.25 × 10^8 ^IU/ml SV/*luc*. **2a: **A typical 2CLP rat showing *luc *activity over right lung and tracheostomy areas (right), left lung and tracheostomy areas (middle), and SO rat showing very low *luc *activity over pulmonary area (left). The color scales values are indicated on the wedge.**2b: **Graphic representation of means and standard deviations of 2CLP + SV/*luc *treated rats. Rt – right lung, Lt – left lung, Trach – tracheostomy area. The values represent the mean for 8 animals and the bars represent standard deviations. The signal over the lung area was different from the signal of tracheal area at P < 0.03 for both left and right lungs.**2c: **Comparison of SV/*luc *and Ad/*luc *transduction (exposure time 2 min) at 48 hours in a rat given intratracheal 300 μl of 1.25 × 10^8^IU/ml SV/*luc *(left) and a rat given intratracheal 300 μl of 1 × 10^9 ^IU/ml Ad/*luc *(right).**2d: **Graphic representation of means and standard deviations of 2CLP + SV/*luc *(8 treated rats, black bar) and 2CLP+Ad/*luc *(3 treated rats, gray bar).

At 24 hours, Luciferase activity was not detected in any animal groups. We explain this finding by the time needed for the reporter gene to express. However, at 48 hours, *luc *activity (detected as luminescence by the CCCD camera) was seen over the lung areas in 2CLP animals given SV/*luc *(Figure 2a). Low levels of *luc *activity were detected in the tracheostomy region, confirming a degree of vector affinity for airway epithelium (Figure 2b). No *luc *activity was detected in rats given PBS. No significant *in vivo luc *activity was detected in the heart, liver, spleen or kidneys of the animals given SV/*luc*.

Fluorescence over the lungs of SO rats was significantly lower than that seen in 2CLP animals (Figure 2a). Intratracheal administration results in little vector uptake in the normal lungs of SO rats, while changes in the intracellular structure occurring during ARDS may be responsible for enhanced viral expression. Similar findings were observed with adenoviruses, and explained by an ARDS-induced exposure/expression of the CAR and integrin receptors that mediate adenoviral entry into the alveolar cells [14]. This mechanism may also apply to the SV40 vector, although different receptors or increased endocytosis may be involved. [39-41].

Based on our previous findings [14,15], a comparison of luc activity between SV/luc and Ad/luc vectors was performed. As expected, luc expression was lower in the 2CLP-SV/luc than in the 2CLP-Ad/luc group (Figure 2c, 2d), because of the natural tropism of adenoviral vectors for the pulmonary epithelium [14]. Another factor may have been the higher titer of the adenoviruses used, a total of 10^9 ^IU/ml adenoviral vector as compared to 1,25 × 10^8 ^IU/ml of the SV40 vector.

### Positive luciferase immunostaining in ARDS lung

**Figure 3:**
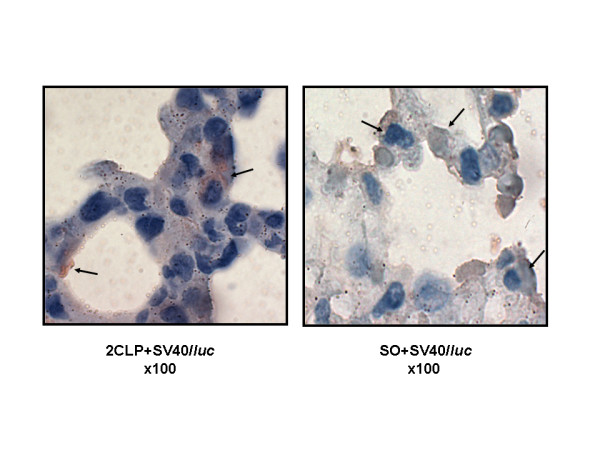
**Luciferase immunostaining**. Was performed on the lung tissue harvested from 2CLP and SO rats given intratracheal SV/*luc*. Positive immunostaining appears as brown intracytoplasmatic coloration (Black arrows). Shown at ×100 magnification.

Luc immunostaining was nonspecific, involving both alveolar type I type II cells. It was moderate in septic animals and low in SO animals (Figure 3). This may be due to moderate infectivity of the SV40 vector in the lungs or weak activity of the SV40 promoter in alveolar cells. This finding is also consistent with the literature, describing less transgene expression of some non – mammalian, non – vertebrate encoded proteins (e.g. luciferase, GFP, lac Z) commonly used as markers for transduction in SV40 vectors, than is usually seen with other vector systems (e.g., adenovirus) [18].

Immunostaining for luciferase in the liver, kidney, spleen and the heart was negative in both 2CLP and SO animals groups given SV/*luc*, suggesting that direct intratracheal administration of the vector results in negligible systemic spread despite alterations in membrane permeability and capillary leak seen in ARDS.

### Positive VP1 immunostaining in ARDS lungs

**Figure 4:**
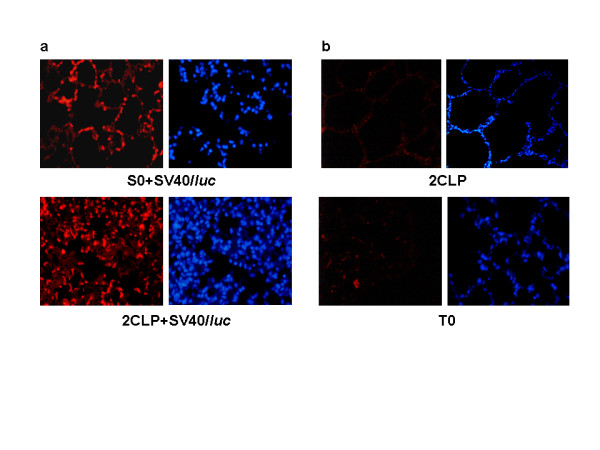
**Detection of the VP1 capsid protein in lung tissue**. **4a**: Positive staining for VP1 protein appears as red intracytoplasmatic coloration: moderate for SO rats (above) and high for 2CLP rats (below). DAPI (blue) was used as nuclear counter-stain. **4b**: Negative control for VP1 immunostaining. Above: lung tissue from control septic rats given intratracheal PBS, and below: normal lung tissue from a control T0 rats. The minimal staining seen represents the background. DAPI (blue) was used as nuclear counter-stain. Shown at ×40 magnification.

In order to test if the low level of Luc immunostaining in lung tissue is due to a low vector penetration into the alveolar epithelium or to low expression of the reporter gene, we tested for the presence of the VP1 capsid protein by fluorescent immunostaining. A significantly higher number of alveolar cells contained viral capsid proteins compared with the number of *luc *positive cells (Figure 4a, 4b, and compare with Figure 3). This suggests that vector penetration into lung epithelial cells is efficient, but transgene expression in many of the cells is below detection level. It is possible that the SV40 promoter used in the present vector is weak and therefore gene expression is lower than gene delivery. A stronger, lung specific promoter may significantly improve expression of a therapeutic gene in alveolar cells.

### The vector infects alveolar type II cells

Alveolar type II cells participate in the regenerative process of the lung. Therefore their transduction is critical for successful ARDS gene therapy.

**Figure 5:**
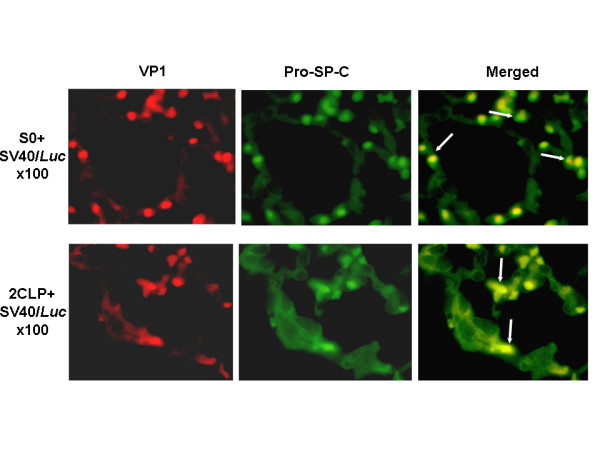
**VP1 detection in type II cells**. The cells were double-stained for VP1 and Pro-SP C as described in Materials and Methods. An SO lung is shown at the top row and ARDS lung in the bottom row. VP1 was detected by Cyte5 (red fluorescence, left panels) and Pro-SP C by FITC (green fluorescence, middle panels). The upper and lower right images show co-localization of both stainings inside alveolar type II cells (yellow). Shown at ×100 magnification

To examine this we performed co-immunostaining of lung tissue for surfactant protein C (Pro SP-C) a marker for alveolar type II cells and VP1 viral capsid protein (Figure 5). The figure depicts co-localization of Pro SP-C and VP1. These findings support our conclusion that the vector is internalized into alveolar type II cells. The ability to transduce these cells is essential because of their importance in alveolar epithelial repair processes and lung recovery in ARDS [10].

### Luciferase mRNA detection

**Figure 6:**
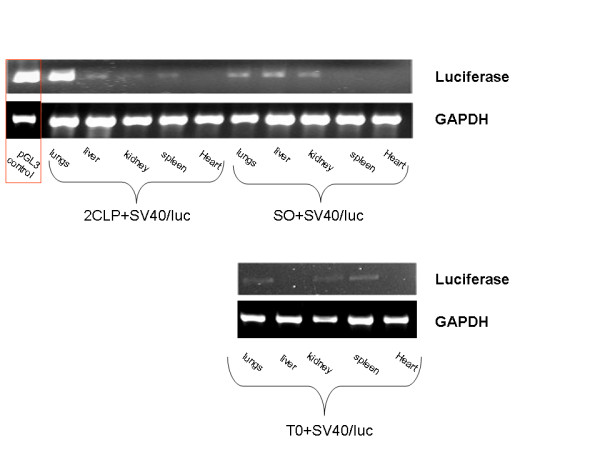
**RT-PCR for luciferase mRNA detection: **High levels of mRNA were detected in the lungs of 2CLP animals (plasmid pGL3 used as positive control for luciferase). Minimal levels were present in the liver, kidney spleen and none in the heart. Minimal expression in the lungs, kidney, liver, spleen, and heart of T0 and SO animals. GAPDH was used as a house keeping control gene, using PCR primers specific for GAPDH.

RT-PCR was performed on frozen tissues of the lung, kidney, liver, spleen and heart. High levels of mRNA were detected in the lungs of 2CLP animals, similar to those found in the plasmid pGL3 used as positive control for luciferase. Minimal levels were present in the liver, kidney spleen and none at all in the heart, confirming minimal systemic spread of the vector after direct intratracheal instillation. Interestingly, in T0 and SO controls, minimal expression was noticed in the lungs, in spite of the same route of vector administration, suggesting that the pathological processes taking place in the sepsis-induced ARDS lungs (as in 2CLP animals) enhances vector penetration and expression by an unknown mechanism. Regarding the kidney, liver, spleen and heart of T0 and SO animals, the same minimal expression was observed as in the septic animals (Figure 6).

### Cellular immune response to SV40 vectors

**Figure 7:**
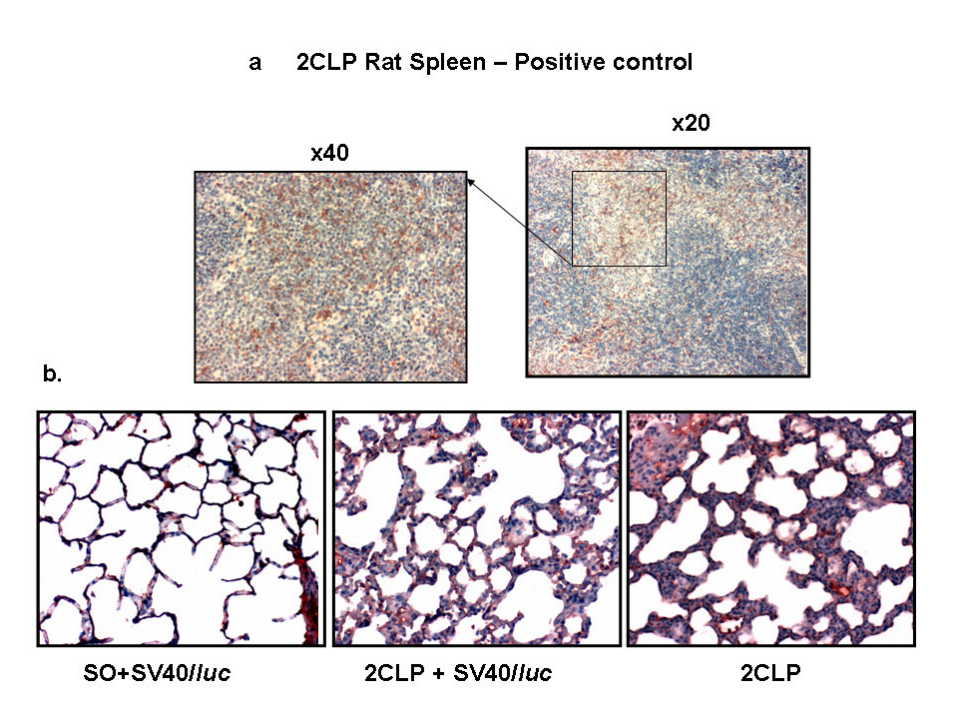
**Immunostaining for CD3+ T cell**. **7a: **Spleen tissue from 2CLP rats serving as positive control for CD3+ T cell immunostainin **7b**: left: minimal lymphocytic infiltration of the lung tissue in SO rat. Middle: moderate infiltration in a 2CLP lung rat after SV/*luc *administration. Right: moderate infiltration in a 2CLP lung rat not treated with SV/*luc*. Note that the degree of lymphocyte infiltration is similar in the middle and right panels, suggesting that it is part of the disease process rather than being induced by the vector.

Direct intratracheal administration of SV/*luc *induced no immunological response as measured by lymphocytic and neutrophilic infiltration of the H&E stained lung sections compared to PBS treated septic lung (Figure 1). To further confirm this we performed immunohistochemical detection for lymphocytic (CD3+ T cells) infiltration (Figure 7a, 7b). As expected, inflammatory cell infiltration was higher in the ARDS compared to the SO lungs. However lymphocytic infiltration in the SV/*luc *and PBS treated septic rats was similar, indicating that the SV40 vector does not elicite an excessive cellular immune response. These findings support our previous results which demonstrated that there was no cellular immune response against the vector or the transgene following liver transduction by the SV/*luc *vector, measured by lymphocyte proliferation assay at 84–110 days following vector administration [30].

Moreover, literature data reported that neutralizing antibody activity against an SV40 vector was not detected in the serum of treated mice, even after 8 consecutive intraperitoneal or subcutaneous inoculations [29]. In our previous study we detected marginal humoral immune response against the vector only at very high vector concentration [30]. Two factors appear responsible of this behavior: the uncommon cell entry route (SV40 vectors evade immune surveillance most likely by entering cells via caveolar endocytosis, followed by vesicular transport directly into the endoplasmic reticulum, bypassing the endosomal-lysosomal pathway) [27], and deletion of T antigen of the viral genome (renders the vector nonimmunogenic) [32].

## Conclusion

The results presented in this article are only preliminary data. Currently, the most common vectors for gene transfer to the lung are replication-deficient adenoviruses. The major advantage of adenovectors is their excellent efficiency in gene transfer. However, gene expression is transient and the immunogenicity prevents repeated administrations. Moreover, the preparation of a gutless adenovirus with a more extended expression is technically demanding [42].

After a detailed search of the literature data we can confirm that this is the first experiment in which an SV40 based vector was used for transduction of lung cells. The goal of our research was to provide a basis for the use of these vectors in face of their advantages over adenoviral vectors, as well as their limitations.

In spite of the two major barriers met by the viruses after intratracheal administration: the mucociliary clearance system and the glycocalix [42], in the present study we have demonstrated efficient uptake of an SV40 vector in the lungs of animals with sepsis-induced ARDS. Although nonspecific, these vectors appear to be capable of *in vivo *transduction of alveolar type II cells, key cells involved in regenerative process of the lung [10].

Acute respiratory disorders like ARDS are the result of a variety of endogenous and exogenous influences, arising rather as a misbalance between protective and destructive mechanisms. Transient gene therapy may thus help restore the homeostatic balance by short term expression of protective genes or suppression of damaging genes [43]. In the near future we plan taking this research a step further by replacing the reporter gene with a therapeutical one (encoding one of the surfactant proteins, Hsp70 or interleukin 6) and trying to alter the course of the disease. SV40 vectors are generally considered non-integrating, transiently expressing vectors [44]. Although others reported a prolonged expression of rSV40 vectors (1 month -1 year) [30], we did not estimate the duration of transgene expression, as our study was limited to 48 hours. Short term or transient gene expression may be sufficient and even advantageous for the treatment of an acute condition like ARDS, where a therapeutic protein or enzyme may be only needed during the acute state. However, as these vectors elicit marginal immune response, repeated administrations, if required, would be possible.

Our results show moderate expression of the luciferase reporter gene. Use of a CMV promoter in the construction of the SV40 vector may improve transgene expression. However, low level of proteins expression may not be entirely disadvantageous. Supra-physiological production of specific proteins may lead to unexpected side effects. In contrast, low expression may be augmented by repeated administrations of the vector [40]. With the SV40 vector repeated administrations are possible due to its low immunogenicity.

Safety considerations require that the vectors do not contain replication associated T antigen sequences. For this reason our vector is T antigen deleted. Vector propagation is achieved in COT18 cells, cell lines with minimal sequence identity with the vector. Reacquisition by rSV40 of T antigen DNA and reemergence of wtSV40 virions remains extremely unlikely [30].

Another major advantage with SV40 vectors is that they may be constructed *in vitro *by packaging DNA of choice in recombinant capsids [45]. These vectors, which combine efficient gene delivery of viral vectors with safety and flexibility of non-viral vectors, were shown to have similar tropism as standard SV40 vectors such as the SV/luc described here [30]. Furthermore, these vectors accommodate plasmids as large as 17 kb, significantly larger than SV40 DNA, and may be therefore used to deliver a variety of genes with complex regulatory signals [46]. Furthermore, these vectors do not require any SV40 sequences, providing additional safety margin [47-49].

The present study suggests that in vitro constructed SV40 vectors may be tailored to deliver genes of choice with lung-specific promoters for treatment of acute respiratory diseases like ARDS.

## Abbreviations

SV40 – Simian virus 40

**SV/*luc ***– Simian virus 40 vector expressing the reporter gene luciferase

Ad – Adenovirus

Ad/*luc *– Adenovirus expressing the reporter gene luciferase

ARDS – Acquired Respiratory distress syndrome

2CLP – Cecal ligation double puncture

SO – Sham operation

UO – Unoperated

PRO-SP-C – Surfactant C protein

VP1 – Viral protein 1

H&E – Hematoxilin eosin

CCCD – Cooling Charged Coupled Device

## Competing interests

A. Oppenheim is a founder of Gene Vector Technology Ltd., whose mission is to develop SV40-based vectors for gene therapy.

## Authors' contributions

The experiment was built on the experience and previous work of Dr.Y. Weiss in adenoviral mediated gene transfer to the alveolar epithelium in sepsis induced ARDS and the expertise of Prof. A. Oppenheim in developing and testing the SV40 vectors in experimental and therapeutic settings. It also relied on an effective method of inducing sepsis in lab rats, which is very similar to what is seen in clinical practice in our ICU units, and which was previously used and developed by Dr.Y. Weiss and his coworkers.

LE – participated in the design of the study and in addition to overall supervision and administration of the project, performed all the animal's experiments and took part in, immunological studies.

ZB – assumed responsibilities for the day-to-day administration, assisted and instructed with the performance of all the pathological studies and immunoassays.

MAL – was in charge of vector preparation, providing the recombinant type needed for this experiment as well as any quantitative or qualitative assays requested for this porpoise.

EZ – helped and instructed with the performance of the *in vivo *measurements using the CCCD camera, as well as data interpretation.

AO – was the consultant expert regarding the SV40 vectors and helped to draft the manuscript.

YGW – conceived the study and participated in its design and coordination and helped to draft the manuscript.

All the authors read and approved the manuscript.
